# Cigarette smoke inhibits LPS-induced FABP5 expression by preventing c-Jun binding to the FABP5 promoter

**DOI:** 10.1371/journal.pone.0178021

**Published:** 2017-05-18

**Authors:** Deviyani Rao, Anne-Laure Perraud, Carsten Schmitz, Fabienne Gally

**Affiliations:** 1 Department of Biomedical Research, National Jewish Health, Denver, Colorado, United States of America; 2 Department of Immunology and Microbiology, University of Colorado Denver, United States of America; Cincinnati Children's Hospital Medical Center, UNITED STATES

## Abstract

Cigarette smoking is the primary cause of chronic obstructive pulmonary disease (COPD) with repeated and sustained infections linked to disease pathogenesis and exacerbations. The airway epithelium constitutes the first line of host defense against infection and is known to be impaired in COPD. We have previously identified Fatty Acid Binding Protein 5 (FABP5) as an important anti-inflammatory player during respiratory infections and showed that overexpression of FABP5 in primary airway epithelial cells protects against bacterial infection and inflammation. While cigarette smoke down regulates FABP5 expression, its mechanism remains unknown. In this report, we have identified three putative c-Jun binding sites on the FABP5 promoter and show that cigarette smoke inhibits the binding of c-Jun to its consensus sequence and prevents LPS-induced FABP5 expression. Using chromatin immunoprecipitation, we have determined that c-Jun binds the FABP5 promoter when stimulated with LPS but the presence of cigarette smoke greatly reduces this binding. Furthermore, cigarette smoke or a mutation in the c-Jun binding site inhibits LPS-induced FABP5 promoter activity. These data demonstrate that cigarette smoke interferes with FABP5 expression in response to bacterial infection. Thus, functional activation of FABP5 may be a new therapeutic strategy when treating COPD patients suffering from exacerbations.

## Introduction

Chronic Obstructive Pulmonary Disease (COPD) is the third leading cause of death in the United States and its global prevalence continues to rise [1]. Although cigarette smoking is by far the most important risk factor of COPD, repeated and sustained infections are clearly linked to disease pathogenesis and are a huge burden on health care costs [2]. Moreover, increased airway and systemic inflammation in stable COPD patients over time is directly linked to disease progression [3]. Thus, reducing inflammation may allow for implementation of appropriate preventive strategies and would be an important approach in the development of new therapies. However, very little is known about the molecular mechanisms whereby cigarette smoke, in conjunction with infections, trigger abnormal and sustained lung inflammation and injury.

Toll-like receptors (TLRs) are pattern recognition receptors that play a pivotal role in mediating the host innate immune response to infections. Specifically, TLR4 recognizes lipopolysaccharide (LPS) [4], a component of Gram-negative bacterial cell walls [5]. TLR4 activation leads to a cascade of signaling events that result in NF-κB and mitogen-activated protein kinases (MAPK) activation. c-Jun, a member of the activator protein 1 (AP-1) family, is then translocated to the nucleus. These signaling events also result in the production of cytokines, chemokines and in the transcription of other genes important for the control of infection [6].

We have recently identified Fatty Acid Binding Protein 5 (FABP5) as an important anti-inflammatory player during respiratory infections. In human airway epithelial cells, FABP5 expression is down regulated by cigarette smoke, thus leading to a more sustained inflammation [7]. In addition, FABP5^-/-^ mice infected with influenza A have heightened lung inflammation that persists long after wild type mice recovery [8]. Although FABP5 protein is abundant in the airways of healthy individuals, it is greatly reduced in the airway epithelium of COPD patients [7, 9]. How *FABP5* gene expression is regulated under basal or diseased conditions is poorly understood. In this study, we dissected in more depth the regulation of FABP5 by cigarette smoke and LPS in airway epithelial cells. We focused on the role of cigarette smoke and/or LPS-mediated MAPK/AP-1 activation in FABP5 regulation, as we identified three putative binding sites for c-Jun within the FABP5 promoter. Understanding how FABP5 expression is regulated during infection will open up novel possibilities to enhance COPD patients’ innate immune defense mechanisms against repeated episodes of infections.

## Materials and methods

### Cell culture and treatment of BEAS-2B cells

The immortalized human epithelial cells isolated from normal human bronchial epithelium obtained from autopsy of non-cancerous individuals, BEAS-2B cells (ATCC CRL-9609) were cultured in BEBM along with all the additives obtained from Lonza/Clonetics Corporation at 37°C, 5% CO_2_. Approximately 75% confluent monolayers of BEAS-2B cells were exposed to cigarette smoke extract and 24 hours later treated with LPS (0.01–1 μg/ml; from *P*. *aeruginosa* serotype 10; Sigma) for up to 48 hours.

### Cigarette smoke extract (CSE)

Two filtered research grade tobacco cigarettes (3R4F) from the Kentucky Tobacco Research and Development Center (University of Kentucky, Lexington, KY) were smoked through an apparatus with a constant airflow (0.2 l/min) driven by an air compressor. The smoke was bubbled through 20 ml of PBS and the pH was adjusted to 7.4. The CSE obtained was filtered and considered 100% CSE. It was further diluted to treat the cells with 2%. The CSE was prepared freshly before each experiment along with an air control extract (AC) consisting of ambient air bubbled into 20 ml of PBS.

### Real Time RT-PCR

RNA was extracted (QIAGEN) and Taqman RNA-to-Ct 1-Step kit (Applied Biosystems) was used to perform Real Time RT-PCR as previously described on GAPDH (Hs03929097_g1, amplicon length 58) and FABP5 (Hs02339439_g1, amplicon length 91) [8]. GAPDH was used as the housekeeping gene. The threshold cycle was recorded for each sample to reflect the mRNA expression levels. The comparative threshold cycle method was used to demonstrate the relative expression level of the gene of interest.

### Western blot

Cells were lysed in RIPA lysis buffer containing protease and phosphatase inhibitors. Ten μg of proteins were electrophoresed on 10% or 15% SDS-PAGE, transferred onto PVDF membrane, blocked with 5% BSA, and then incubated with a rat anti-FABP5 antibody (Clone # 311215, R&D systems), a rabbit anti-c-Jun antibody (Cell signaling), a rabbit anti-phosphoSer63-c-Jun antibody (Cell signaling), or a rabbit anti-phosphoSer73-c-Jun antibody (Cell Signaling) overnight at 4°C. After washes in PBS with 0.1% Tween-20, the membranes were incubated with anti-rat or anti-rabbit IgG conjugated to horseradish peroxidase for FABP5, c-Jun and phospho-c-Jun protein detection. Membranes were stripped and re-probed with a mouse anti-GAPDH antibody (6C5 sc-32233, Santa Cruz Biotechnologies). Densitometry was performed to quantify proteins of interest.

### c-Jun activity assay

Cells were homogenized in nuclear protein extraction buffer to extract nuclear proteins following manufacturer’s instructions (Active Motif, Carlsbad, CA). Nuclear proteins (20 μg per sample) were used to perform c-Jun ELISA (Active Motif, Carlsbad, CA) to quantify c-Jun activation. Briefly, multiple copies of c-Jun specific double-stranded oligonucleotide were immobilized to a 96-stripwell plate. Cell nuclear extracts (protein) were then added to the well where activated c-Jun bound specifically to the oligonucleotide at its consensus-binding site. Thereafter, a primary antibody specific for the activated form of c-Jun was added, followed by incubation with HRP-conjugated secondary antibody and then developing solution. Results were expressed as absorbance read at 450nm with a reference wavelength at 650nm.

### Chromatin immunoprecipitation (ChIP) assay

BEAS-2B cells were exposed to cigarette smoke extract or air control for 24 hours and then stimulated with LPS for 1 hour. The ChIP assay was performed using a ChIP assay kit (Active Motif, Carlsbad, CA) according to the manufacturer’s specifications. Immunoprecipitation was carried out overnight at 4°C with 2 μg of c-Jun antibody (Active Motif, Carlsbad, CA). Polymerase chain reaction (PCR) was performed with input DNA (5μl, diluted 1:10) and immunoprecipitated DNA (5 μl) using FABP5 promoter-specific primers: forward 5’-TTGTGCGCTGGCGAGG-3’, reverse 5’-CCCTCGCCGGACTCGG-3’. The PCR products were subjected to electrophoresis on 2% agarose gels containing ethidium bromide. The expected PCR product is 104 bp.

### FABP5 promoter construct, site-directed mutagenesis and luciferase reporter assay

Based on the sequence provided for the *FABP5* gene (accession number NG_028154) and using MacVector, we designed the following primers to clone the FABP5 promoter from BEAS-2B genomic DNA by polymerase chain reaction: forward 5’-CCGCTCGAGCTTTTAATCCAAAGTAAG-3’, reverse 5’-CCTAGGCCAATTCATGTATTCATC-3’ into TOPO vector (Invitrogen). The expected PCR product size is 5,143 bp with the inclusion of an XhoI and EcoRI restriction sites in the forward and reverse primers, respectively. The promoter was then subcloned into pGL4.18 vector (Promega, Madison, WI). Site-directed mutagenesis was used to mutate the c-Jun binding site by polymerase chain reaction amplification (QuikChange site-directed mutagenesis kit; Stratagene, La Jolla, CA). The following primers were used to create the mutation in the c-Jun binding site at position -495 on the FABP5 promoter (accession number NG_028154) with two T substitutions for two A: forward 5’-CCGTAGTTAATGTATCTATAAACC-3’ and reverse 5’-GGTTTATAGATACATTAACTACGG-3’. Plasmid DNAs were amplified, confirmed by restriction analysis and by sequencing. BEAS-2B cells were co-transfected with a firefly luciferase reporter gene (F-luc) containing FABP5 promoter sequence (-5072bp/+80bp) (pGL4-FABP5) and renilla luciferase (R-luc) construct (pGL4-CMV) at a 10 to 1 ratio. The cells were then exposed to cigarette smoke or air control and stimulated the following day with LPS (1μg/ml) for 2 hours. Firefly luciferase (F-luc) and renilla luciferase (R-luc) activity were determined using a dual luciferase reporter assay kit (Promega, Madison, WI). The transfection efficiency was normalized by the ratio of F-luc and R-luc to determine changes in FABP5 promoter activity.

### Data analysis

Data are expressed as mean ± SEM. One-way analysis of variance was used for multiple comparisons, and Tukey's post hoc test was applied where appropriate. Student's *t* test was used when only two groups were compared. Differences were considered statistically significant when *p* < 0.05.

## Results

### Validation of the BEAS-2B human lung epithelial cell model system

**Fig 1:**
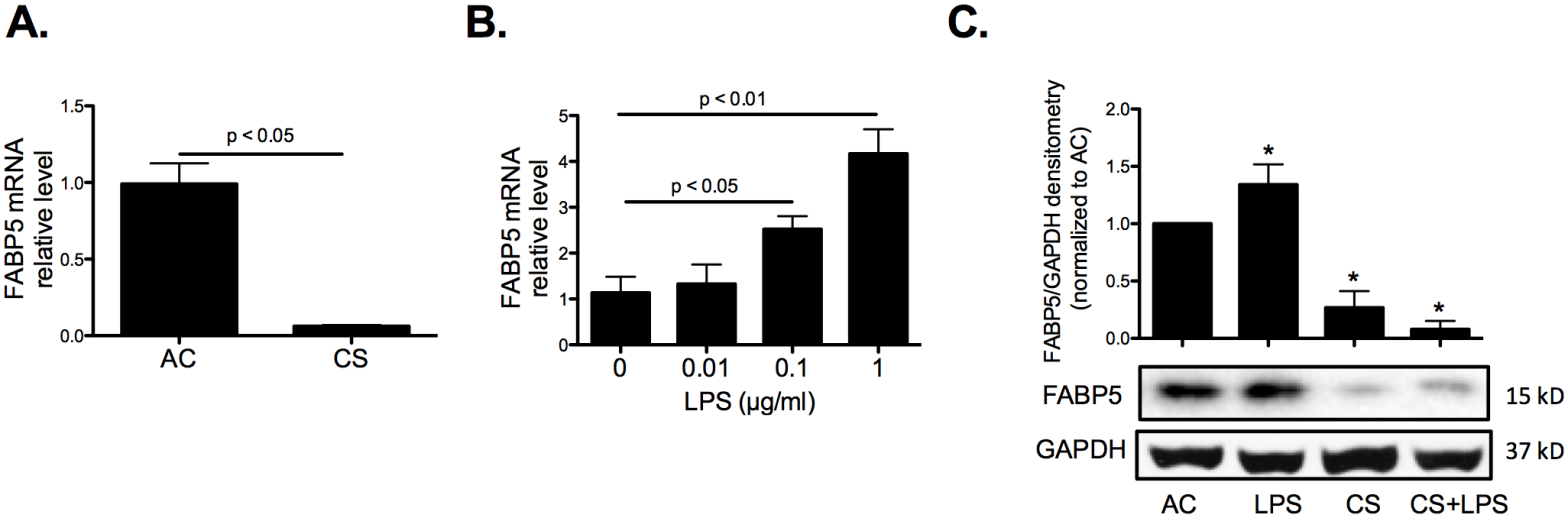
CS inhibits FABP5 expression whereas LPS increases FABP5 expression in human lung epithelial BEAS-2B cells. **A.** Cells were exposed to 2% cigarette smoke extract (CSE) for 48 hours. FABP5 mRNA expression was determined by real-time PCR. **B.** Cells were exposed to LPS derived from *P*. *aeruginosa* at the indicated concentrations for 48 hours. FABP5 mRNA expression was determined by real-time PCR. **C.** Cells were exposed to 1 μg/ml LPS derived from *P*. *aeruginosa* and/or 2% CSE for 48 hours. FABP5 protein expression was determined by Western blot. Data are representative of 3 independent experiments. * p<0.05.

We have previously shown that cigarette smoke inhibits FABP5 expression whereas *P*. *aeruginosa* infection increases FABP5 expression in primary normal human airway epithelial cells [7]. In order to perform molecular expression studies, we needed to use a cellular model and chose the BEAS-2B human lung epithelial cell line. To validate this cell culture model, we exposed BEAS-2B cells to 2% cigarette smoke extract (CSE) for 48 hours and measured FABP5 mRNA expression. As shown in Fig 1A, CSE greatly reduced FABP5 mRNA expression. In contrast, cells exposed to LPS derived from *P*. *aeruginosa* showed increased levels of FABP5 mRNA expression in a dose-dependent manner (Fig 1B). Likewise FABP5 protein expression was enhanced in human lung BEAS-2B epithelial cells following treatment with 1 μg/ml LPS for 48 hours, but greatly reduced in the presence of 2% CSE (Fig 1C). Taken together, these results indicate that the human lung epithelial BEAS-2B cells behave similarly to primary human cells regarding *FABP5* gene expression, and therefore are an adequate model to study the regulation of FABP5 expression by cigarette smoke and/or infection.

### LPS increases c-Jun activity in BEAS-2B cells

**Fig 2:**
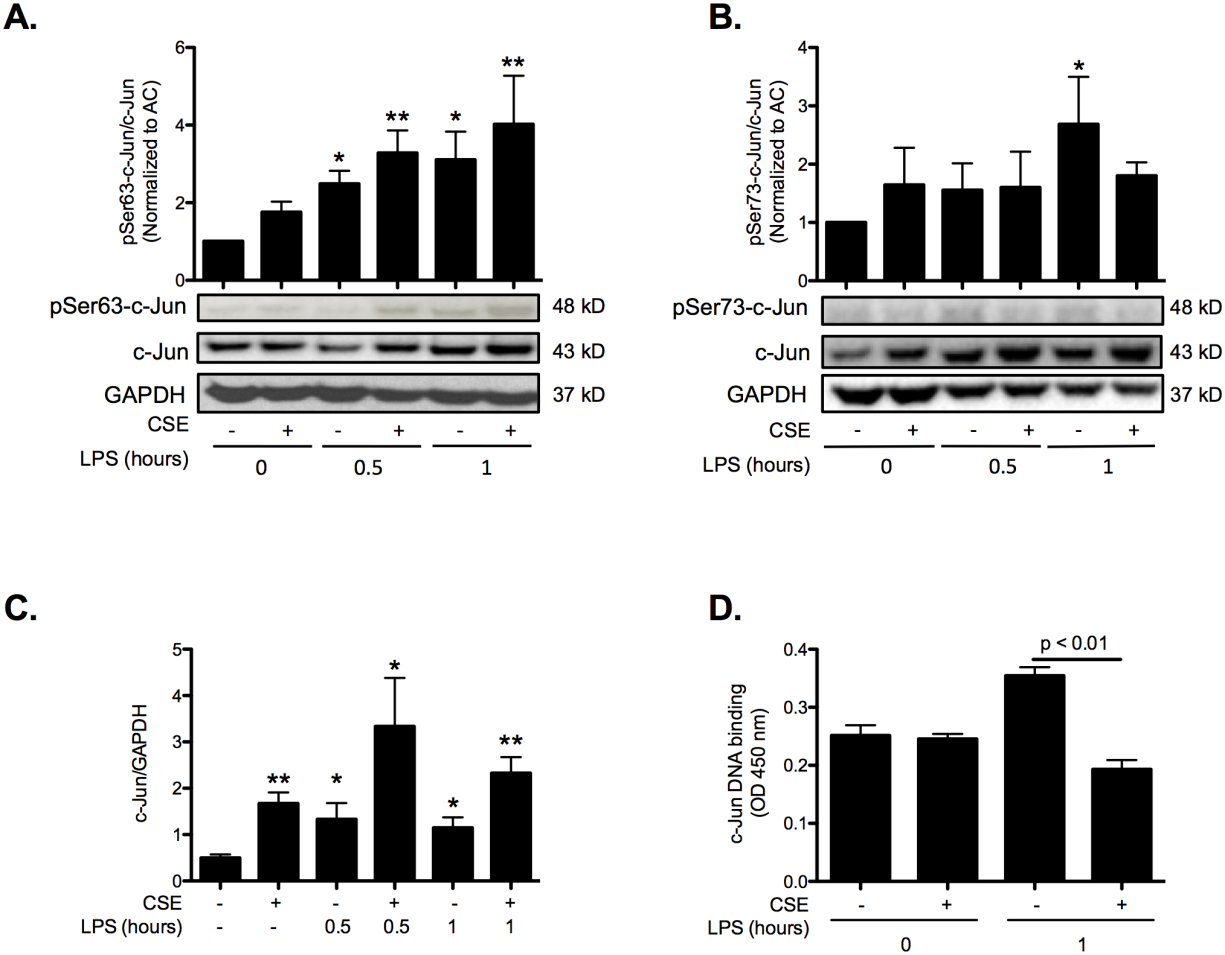
CS inhibits c-Jun activity in human lung epithelial BEAS-2B cells. Cells were treated with 2% CSE for 24 hours and then stimulated with 1 μg/ml LPS for the indicated time points. **A.** Phosphorylation of c-Jun at Ser63 and total c-Jun were determined by Western blot. **B.** Phosphorylation of c-Jun at Ser73 and total c-Jun were determined by Western blot. **C.** Ratio of total c-Jun and GAPDH densitometry determined by Western blot. **D.** Activation of c-Jun transcription factor was determined using an ELISA-based TransAM c-Jun activation assay. Data are representative of 6 independent experiments. * p<0.05, ** p<0.01 compared to no CSE and no LPS.

At 1 μg/ml, LPS maximally induced FABP5 gene (4-fold) and protein (1.5-fold) levels (Fig 1B and 1C); therefore, this dose was chosen in subsequent experiments. Because the AP-1 transcriptional system is a well-characterized downstream target of TLR4 signaling, and c-Jun is one of the subunit of AP-1, we next confirmed that LPS treatment led to c-Jun phosphorylation in BEAS-2B cells. The transcriptional activity of c-Jun is regulated by phosphorylation at Ser63 and Ser73 through SAPK/JNK [10]. Both LPS and CSE led to increased c-Jun phosphorylation at Ser63. A maximal increase of c-Jun phosphorylation at Ser63 was observed in CSE-treated cells after 1 h of LPS exposure (Fig 2A). Phosphorylation of c-Jun at Ser73 was only significantly induced after 1 h of LPS exposure in the absence of CSE (Fig 2B). Interestingly, both LPS and CSE increased c-Jun protein amount. The combined treatment did not however increased c-Jun protein amount any further than CSE alone (Fig 2C). Previous studies have shown that phosphorylation of c-Jun at Ser73 in the N-terminal transactivation domain positively regulates c-Jun DNA binding activity [11]. Therefore, we examined the DNA binding activity of the total nuclear extract of BEAS-2B CSE-exposed and/or LPS-treated cells to a generic c-Jun consensus sequence. After 1 or 2 hours of LPS treatment, c-Jun binding to its generic consensus sequence increased. However, LPS-induced c-Jun binding was back to basal level if the cells were pre-treated with 2% CSE (Fig 2D). Taken together these data indicate that CSE does not induce c-Jun phosphorylation at Ser73, thus preventing c-Jun binding to its consensus sequence.

### LPS enhances, while CSE impairs, c-Jun binding to the FABP5 promoter

**Fig 3:**
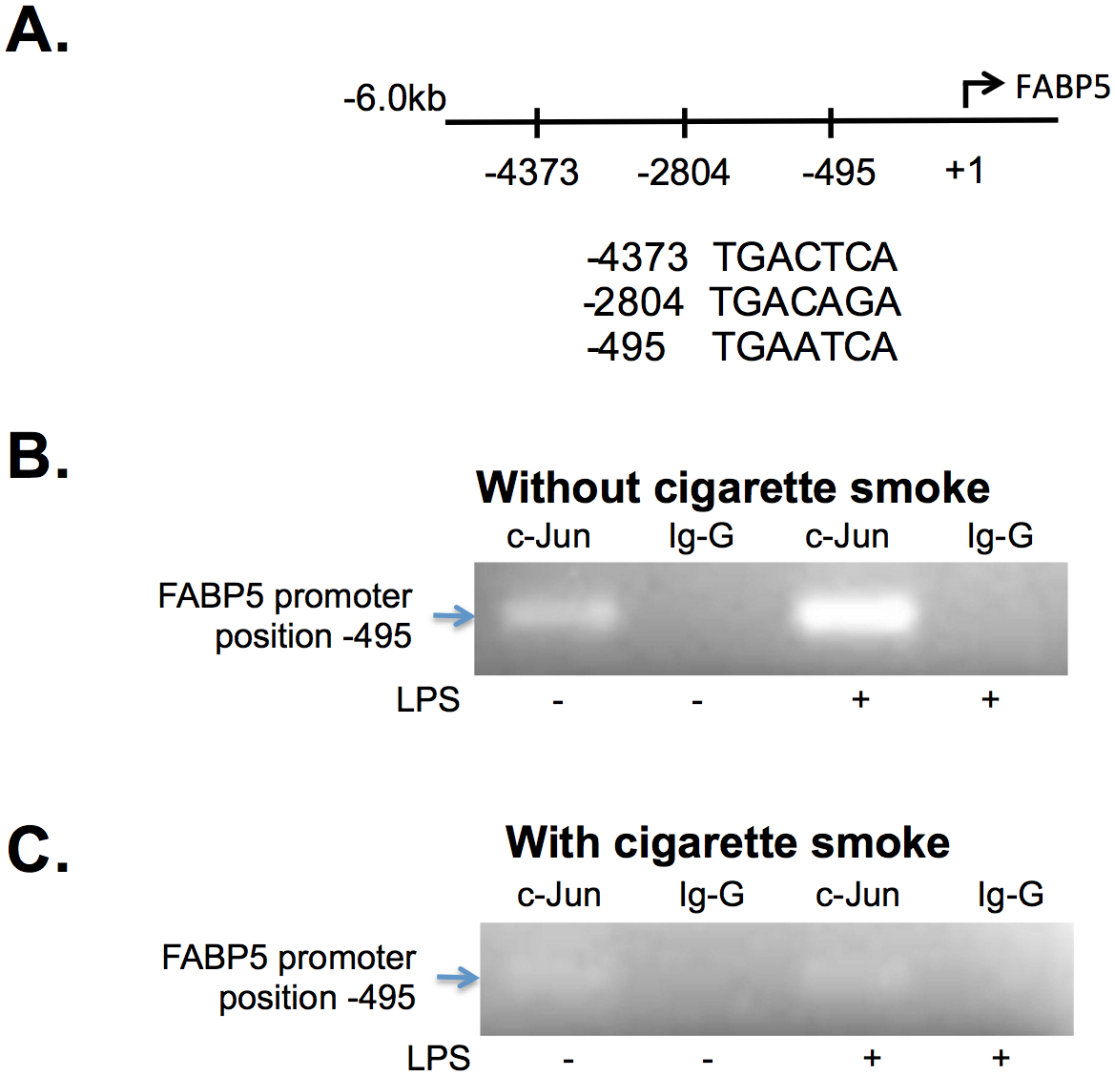
CS inhibits c-Jun binding to the FABP5 promoter in human lung epithelial BEAS-2B cells. **A.** Schematic of the three putative c-Jun binding sites on the *FABP5* promoter region (not drawn in scale). **B.** Without and **C.** with 2% CSE for 24 hours. Cells were stimulated with LPS (1 μg/ml) for 1 hour and chromatin immunoprecipitation assays were performed using anti-c-Jun or anti-rabbit Ig-G antibodies. PCR was performed using primers specific to the proximal c-Jun consensus site (-495) within the *FABP5* gene promoter. Data are representative of 3 independent experiments.

Bioinformatics analysis using TESS (transcription element search software) revealed three putative c-Jun binding motifs in the 5’ flanking region (-5 kb) from the FABP5 transcription start site (positions -4373, -2804, -495) (Fig 3A). To study the functional relevance of these sites, we performed chromatin immunoprecipitation (ChIP) assays on CSE-exposed and/or LPS-treated BEAS-2B cells using c-Jun and the corresponding Ig-G antibody as a negative control. Interestingly, c-Jun was increasingly recruited to the proximal promoter region (position -495) after LPS stimulation (Fig 3B). The other two sites (position -4373 and -2804) did not show any recruitment of c-Jun with or without LPS (data not shown). The presence of CSE greatly reduced the recruitment of c-Jun to this site (Fig 3C). These findings not only demonstrate that there is an association between LPS-induced c-Jun recruitment to the *FABP5* gene promoter, causing enhanced FABP5 mRNA and protein accumulation, but also that cigarette smoke can interfere with FABP5 expression in response to bacterial infection.

### LPS increases, while CSE or mutation of the c-Jun binding site decreases, the transcriptional activity of the FABP5 promoter

**Fig 4:**
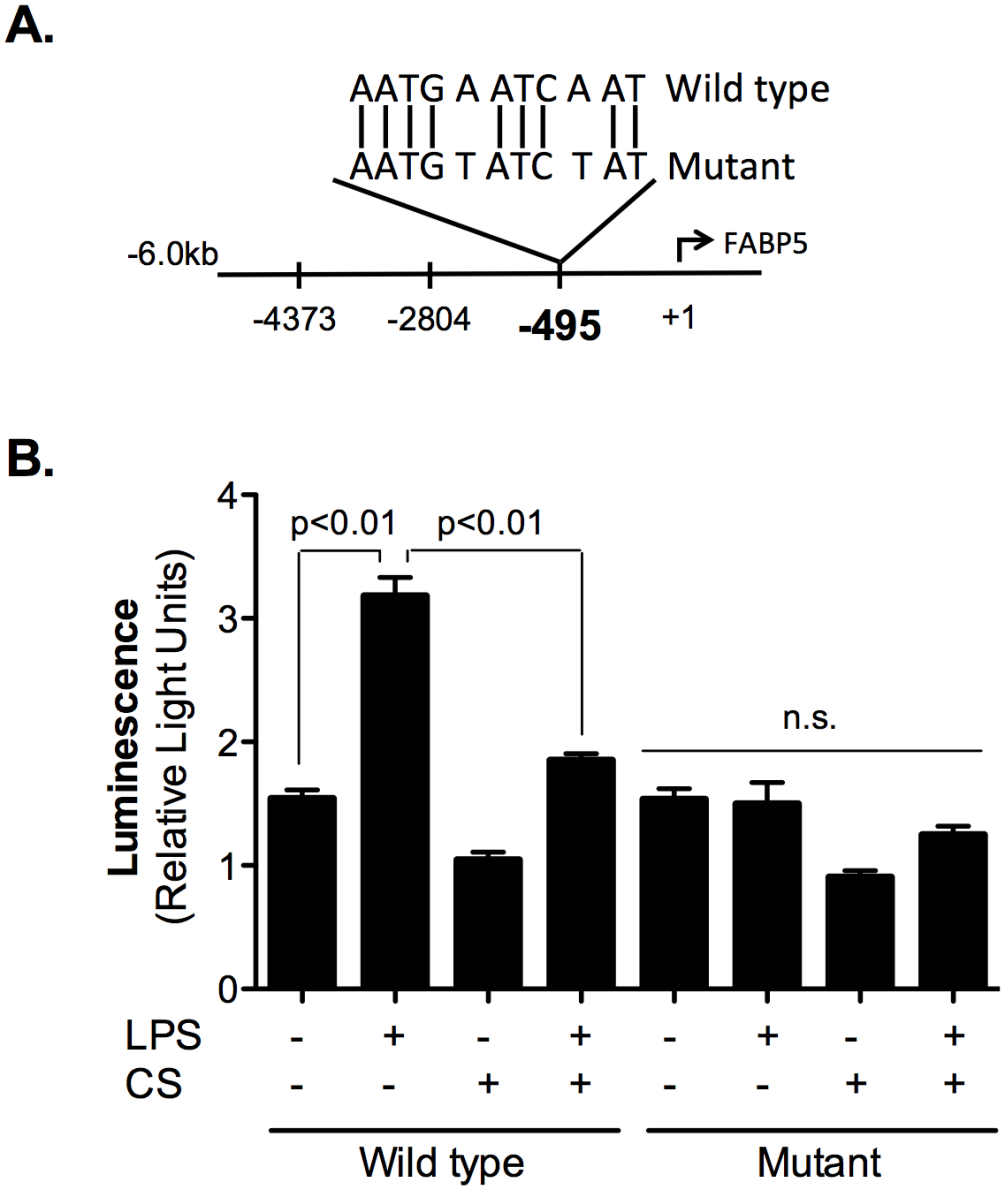
CS inhibits *FABP5* promoter activity in human lung epithelial BEAS-2B cells. **A.** Schematic overview of the wild type and mutant c-Jun binding site on the FABP5 promoter at position -495. **B.** Dual luciferase assay following 2% CSE and/or 1 μg/ml LPS treatment of the wild type *FABP5* promoter or the promoter mutated at position -495 in the c-Jun binding site. Data are representative of 3 independent experiments.

BEAS-2B cells were transiently transfected with an FABP5 promoter reporter construct and renilla luciferase as transfection control. Either wild type or mutated FABP5 promoter construct at position -495 were used, as illustrated in Fig 4A. The next day, cells were exposed to CSE for 24 hours and then treated with LPS for 2 hours. LPS treatment significantly increased FABP5 promoter activity compared to untreated samples (Fig 4B). However, when cells were pre-treated with CSE, we did not observe a significant increase in FABP5 promoter activity following LPS stimulation (Fig 4B). Similarly, when the -495 c-Jun binding site on FABP5 promoter was mutated, we did not observe any increase in FABP5 promoter activity following LPS treatment (Fig 4B). Together our data suggest that CSE impairs c-Jun binding to the FABP5 promoter.

## Discussion

COPD is a complex disease characterized by an abnormal lung inflammatory response to cigarette smoke that leads to tissue destruction and airflow obstruction [12]. Acute exacerbations, mainly caused by bacterial and viral infections, are a major cause of hospitalization and death which can be a serious burden on health care costs [13] as well as patient quality of life. It has been shown that COPD patients are more susceptible to infections than non-COPD smokers [14–16]. Smoking appears to dampen innate immune responses, and as a consequence, pathogens proliferate and persist in the airways of COPD patients [17]. Exposure to tobacco smoke increases the risk of pulmonary infection [18]. Animal studies have shown that smoke exposure results in decreased host defense of the lung [19, 20]. Specifically, cigarette smoke impairs mucociliary clearance [21], enhances bacterial adhesion to airway epithelium [22], compromises the immune system [23], inhibits epithelial cells host defense function [24], and thus increases susceptibility to infections [20, 25, 26]. Bacterial colonization of the airways of smokers may contribute to COPD disease progression and may be responsible for the irreversible pulmonary tissue damage in ex-smokers. For example, cigarette smoke extract suppressed LPS-induced AP-1 activation in bronchial epithelial cells [27]. However, the mechanisms behind the immunosuppressive effect of cigarette smoke and consequent bacterial colonization are poorly understood.

We have previously shown that bacterial infection and cigarette smoke exposure differentially regulate FABP5 expression [7, 9], however little is known about the molecular mechanisms of *FABP5* gene regulation. Our data are showing, for the first time, that LPS increases FABP5 transcript expression in a dose-dependent manner in BEAS-2B lung epithelial cells. We also demonstrate that c-Jun, a subunit of the activator protein 1 (AP-1) transcription factor, binds to a consensus sequence we identified in the FABP5 promoter region. In addition, we established that cigarette smoke impairs c-Jun binding to the FABP5 promoter, providing a molecular mechanism underlying our observation that CSE negatively affects FABP5 expression. Therefore, our experiments with the BEAS-2B lung epithelial cell line suggest that LPS-induced FABP5 up-regulation may be part of an effective innate host response aimed at protecting lung tissue against bacterial infection-induced inflammation. Further *in vivo* studies will be needed in the future to confirm the physiological impact of our findings.

The putative interaction of *FABP5* with AP-1 was previously reported [28] when the murine *FABP5* gene was cloned, along with other transcription factors including myogenic differentiation factor (MyoD), C/EBPα and β, HNF1, myeloid zinc finger 1 (MZF1) and GATA1. The authors identified 560 binding sites for 40 different transcription factors with a level of 80% identity to the consensus sequence, and suggested that the transcription factors might be involved in regulating the expression pattern of FABP5 [28]. In addition, cooperation between FABP5 and PPARβ/δ has been shown to enhance cell survival and proliferation in prostate cancer cells [29]. FABP5 is thought to deliver ligands to the nuclear receptor PPARβ/δ, thus enhancing its transcriptional activity. In turn, FABP5 expression is up-regulated by PPARβ/δ activity, indicative of a positive feedback loop between the two [29]. Thus, FABP5 gene expression is highly regulated by different transcription factors that may play different roles depending on the tissue or the stimulus being considered. Whether these transcription factors affect one another is a possibility that needs to be investigated in future experiments.

Being at the interface between the host and the environment, the airway epithelium represents the first line of host defense against potential pathogens or irritants such as cigarette smoke. This barrier has to not only serve the physiological function of the lung, but also protect its well-being. The protective role of the epithelium includes recognition [30] and response to potentially dangerous particulates and microbes by producing various mediators such as proinflammatory cytokines (GM-CSF and IL-8), mucins (MUC5AC) and antimicrobial substances including peptides (β-defensins and LL-37) and proteins (lysozyme and lactoferrin) [31]. Production of these inflammatory cytokines and anti-microbial substances is controlled by different transcription factors (e.g. NF-κB and AP-1). However, the persistent and repeated nature of infections within COPD patients indicates an abnormal epithelial cell function.

Airway TLR signaling initiates inflammatory responses and increases the production and secretion of host defense molecules that collectively provide protection against respiratory infections [32]. We have shown previously that while *Mycoplasma pneumoniae* or *Pseudomonas aeruginosa* infections increase FABP5 expression in primary human airway epithelial cells, cigarette smoke reduces FABP5 expression [7, 9]. The results presented here, using the immortalized human lung epithelial cell line BEAS-2B, are in agreement with our previous findings in primary human airway epithelial cells [7], thus, validating BEAS-2B cells as a good model to study *FABP5* gene regulation. Furthermore, we chose BEAS-2B cells for their sturdier nature and easier manipulation, especially for transfection of FABP5 promoter reporter construct, rather than primary human airway epithelial cells.

We observed up to 33% increase in FABP5 promoter activity after only 2 hours of LPS treatment indicating that FABP5 transcription is a rapid event following c-Jun activation, which also appears after 2 hours of LPS treatment. On the contrary, cigarette smoke exposure for 24 hours completely suppressed FABP5 promoter activity and gene transcription. In addition, we have shown that site-directed mutagenesis (at position -495) to disrupt c-Jun binding site was sufficient to reduce FABP5 promoter activation following LPS treatment.

FABP5, a novel anti-inflammatory protein, is significantly reduced in inflammatory lung diseases including COPD [7, 9], which may render the patients more susceptible to bacterial infections. This study advances our understanding of *FABP5* gene regulation and may open up new therapies for the treatment and/or prediction of recurrent exacerbations that are triggered primarily by bacterial and viral infections and are associated with increased airway inflammation.
